# Positive Airway Pressure-Related Aerophagia in Obstructive Sleep Apnea: Results from the InterfaceVent Real-Life Study

**DOI:** 10.3390/jcm14186424

**Published:** 2025-09-11

**Authors:** Celia Vidal, Jean-Pierre Mallet, Sarah Skinner, Raphael Gilson, Olivier Gaubert, Arnaud Prigent, Frédéric Gagnadoux, Jean-Christian Borel, Arnaud Bourdin, Nicolas Molinari, Dany Jaffuel

**Affiliations:** 1 Desbrest Institute of Epidemiology and Public Health, National Institute of Health and Medical Research, Montpellier University, 34090 Montpellier, France; nicolas.molinari@inserm.fr; 2 Groupe Adène, 34080 Montpellier, France; jp-mallet@chu-montpellier.fr (J.-P.M.); r.gilson@groupe-adene.com (R.G.); o.gaubert@groupe-adene.com (O.G.); a-bourdin@chu-montpellier.fr (A.B.); dany.jaffuel@wanadoo.fr (D.J.); 3 Department of Respiratory Diseases, Montpellier University Hospital, Arnaud de Villeneuve Hospital, 34090 Montpellier, France; 4 PhyMedExp (National Institute of Health and Medical Research U 1046, National Center for Scientific Research UMR9214), Montpellier University, 34090 Montpellier, France; 5 Service de l’Information Médicale, Epidémiologie et Données de Santé, Montpellier University Hospital, 34295 Montpellier, France; sarah.skinner@chu-montpellier.fr; 6 Polyclinique Saint-Laurent, Groupe Medical de Pneumologie, 35700 Rennes, France; dr.arnaudprigent@gmail.com; 7 Department of Respiratory and Sleep Medicine, University Hospital of Angers, 49100 Angers, France; frgagnadoux@chu-angers.fr; 8 Centre de Pneumologie Henri Bazire, 38134 La Sure en Chartreuse, France; jcborel1@gmail.com; 9 PreMEdical-National Institute for Research in Digital Science and Technology, Montpellier University Hospital, 34295 Montpellier, France

**Keywords:** CPAP, aerophagia, side effect, OSA

## Abstract

**Background:** Continuous positive airway pressure (CPAP) effectiveness can be compromised by adverse effects. Despite its potential impact on adherence and sleepiness, aerophagia remains under-recognized and poorly characterized. This ancillary analysis of the InterfaceVent study aimed to identify risk factors for aerophagia in a large real-life cohort of CPAP-treated patients and to assess its association with both CPAP adherence and sleepiness. **Methods:** InterfaceVent was a prospective, real-life, cross-sectional study. Adults treated for at least 3 months with CPAP were included. Patients self-reported mask-related side effects using visual analogue scales. Aerophagia was defined as a dichotomous outcome based on patient-reported symptoms and CPAP non-adherence as mean nightly usage <4 h. Sleepiness was assessed using Epworth Sleepiness Scale (ESS). **Results:** A total of 1461 patients (median age 67 years (Q1–Q3; 60–74); 27.6% women) were included. Aerophagia was reported by 8.3% of participants. Compared to patients without aerophagia, those affected were younger, more frequently female, and had lower BMI. Patients with aerophagia reported a median ESS score of 7 (4–10) versus 5 (3–8) for patients without aerophagia (*p* < 0.001). CPAP usage was significantly lower in the aerophagia group (median 6.37 vs. 6.75 h/day; *p* = 0.001), whereas non-adherence, did not significantly differ between groups (10.7% vs. 7.5%; *p* = 0.20). **Conclusions:** This ancillary analysis of the InterfaceVent study highlights the burden of aerophagia in CPAP-treated patients and identifies modifiable and non-modifiable risk factors. Better recognition and management of this under-reported side effect may improve CPAP adherence and patient comfort. **Trial registration:** InterfaceVent is registered with ClinicalTrials.gov (NCT03013283). The first registration date is 23 December 2016.

## 1. Background

Continuous positive airway pressure (CPAP) remains the cornerstone therapy for obstructive sleep apnea (OSA), demonstrating high efficacy in reducing apneas, improving daytime symptoms, and mitigating cardiovascular risk [1,2,3]. However, in real-life settings, the effectiveness of CPAP may be compromised by treatment-related side effects that reduce comfort and limit adherence [4]. Aerophagia, in particular, remains poorly understood and often overlooked, despite its potential impact on patient acceptance and long-term use.

The current published literature suggests that the prevalence of CPAP-related aerophagia is consistently at or above 7.2% [5], highlighting that this side effect is likely under-recognized. Although limited research has investigated the risk factors associated with CPAP-related aerophagia, studies have identified significant associations between aerophagia and higher CPAP pressure levels [5,6], gastroesophageal reflux disease [5,7,8], reflux medications [7,8], younger age, and lower body mass index (BMI) [9].

The InterfaceVent study, a large prospective observational study of long-term CPAP users, previously investigated the impact of mask-related side effects on CPAP adherence and residual sleepiness [4]. Although aerophagia was observed in 8.3% of participants, it was not specifically addressed in the initial analysis. Utilizing the same cohort, we conducted a dedicated ancillary analysis to better characterize CPAP-related aerophagia and identify its clinical and technical determinants.

## 2. Methods

### 2.1. Study Design and Study Population

This ancillary analysis is part of the InterfaceVent study [4]. The InterfaceVent study (ClinicalTrials.gov: NCT03013283) was a prospective, real-life, cross-sectional study in adults undergoing at least 3 months of CPAP. Patient enrolment took place between 7 February 2017 and 1 April 2019. We report here the results for patients with Sleep Apnea Syndrome (SAS) treated exclusively with CPAP. SAS was defined according to the criteria established by the French Social Security (FSS) system: (i) an Apnea–Hypopnea Index (AHI) ≥ 30 events/h or an AHI ≥ 15 events/h in combination with more than 10 respiratory-effort-related arousals per hour and (ii) the presence of excessive daytime sleepiness together with at least three clinical features among snoring, morning headaches, hypertension, impaired vigilance, reduced libido, or nocturia. Fulfillment of these criteria is a prerequisite for reimbursement approval by the FSS. Exclusion criteria comprised ongoing pregnancy plans, breastfeeding, inability to comprehend the study’s purpose or communicate with the investigator, concurrent participation in another trial involving an exclusion clause, lack of affiliation with the French Social Security system, any form of legal guardianship or judicial protection, and the use of an intra-oral device or more than one mask. Patient inclusion was carried out during a scheduled home visit conducted by one of 32 technicians from Apard (Montpellier, France), a non-profit home care provider. These visits were scheduled in accordance with the follow-up requirements set by the French Social Security system for CPAP reimbursement. Patients had unlimited access to 34 masks.

### 2.2. Collected Data

For each participant, a single data collection was conducted. Demographic characteristics, mask-related side effect visual analogue scale scores, Epworth Sleepiness Scale results, and EQ-5D-3L questionnaire responses were obtained exclusively on the inclusion date. Aerophagia was defined as a binary variable based on the presence of patient-reported symptoms such as abdominal bloating, flatulence, or eructation, collected during routine technician follow-up visits. No validated questionnaire was available in the dataset, and no distinction was made between new-onset symptoms and those predating CPAP initiation.

The Epworth Sleepiness Scale, the EQ-5D-3L questionnaire, and visual analogue scales (VAS) used to assess mask-related side effects were administered by a technician employed by the home care provider during a scheduled visit. The 11-point VAS (0 = no reported side effects; 10 = very uncomfortable side effects) assessed the following mask-related side effects: dry mouth, partner disturbance due to leaks, patient-reported leaks, noisy mask, heavy mask, painful mask, mask injury, painful harness, harness injury, redness of the eyes, itchy eyes, dry nose, stuffy nose, and runny nose. Importantly, the technician did not help patients fill out the questionnaires or the VAS.

### 2.3. Statistical Analyses

Continuous data were expressed as medians with their associated interquartile ranges due to non-Gaussian distributions. Age was categorized into terciles to improve the interpretability of results. Qualitative parameters were expressed as numbers and percentages. The effect of aerophagia was evaluated using Wilcoxon–Mann–Whitney tests for quantitative data and Chi-square or Fisher’s exact tests for qualitative data. Side effects, according to aerophagia, were compared using Chi-square or Fisher’s exact tests. For these last comparisons, *p*-values were adjusted using the Benjamini–Hochberg False Discovery Rate (FDR) correction method. Univariate and multivariable logistic regression analyses were used to explore associations between patient characteristics and the occurrence of aerophagia. First, univariate analyses were conducted for all potential explanatory variables listed in Table 1. All variables in univariate analyses were included in multivariable analyses using backward selection. CPAP non-adherence was defined as a mean nightly CPAP usage of less than 4 h. All statistical analyses were performed with R (V.4.3.1).

## 3. Results

Table 1 presents the baseline characteristics of the study population. A total of 1461 adults were included in the analysis, of whom 8.3% (*n* = 121) reported experiencing aerophagia.

These patients were younger (median age 63 vs. 67 years, *p* < 0.001) and more likely to be female (41.3% vs. 26.4%, *p* < 0.001). They had a lower BMI (30.1 vs. 30.8 kg/m^2^, *p* = 0.024) and were more likely to be active workers (37% versus 19%, *p* < 0.001). Patients with aerophagia reported higher daytime sleepiness compared to those without, with a median Epworth Sleepiness Scale score of 7 versus 5 (*p* < 0.001). CPAP usage was lower in the aerophagia group, with median usage of 6.37 vs. 6.75 h/day (*p* = 0.001), whereas non-adherence did not significantly differ between groups (aerophagia = 10.7% vs. no aerophagia = 7.5%; *p* = 0.20). Patients with aerophagia tended to receive higher mean CPAP pressures than those without (median 8.5 vs. 8.1 cm H_2_O), although this difference was not statistically significant (*p* = 0.111). However, the distribution across pressure categories revealed significant differences; nearly one-third of patients with aerophagia (29.4%) were treated in the 10–12 cm H_2_O range, compared to only 20.4% of those without (*p* = 0.018). No significant differences were observed in the distribution of 90th/95th percentile pressure values between groups (median 10.3 vs. 10.0 cm H_2_O; *p* = 0.062). Among patients treated with fixed-pressure CPAP, the proportion of those reporting aerophagia was lower (10.7%) compared to those without aerophagia (13.2%), although this difference was not statistically significant (*p* = 0.438). Patients treated with fixed-pressure CPAP had significantly higher mean pressure levels compared to those using auto-CPAP yet paradoxically exhibited lower 90th/95th percentile pressure values (9.29 ± 2.09 vs. 8.26 ± 2.19 cm H_2_O for mean pressure; 9.3 ± 2.09 vs. 10.10 ± 2.26 cm H_2_O for 90th/95th percentile pressure; both *p* < 0.001).

Unintentional leaks were higher in patients without aerophagia (median 2.5 L/min vs. 1.2 L/min, *p* = 0.036). Use of heated humidifiers and/or heated breathing tubes was more frequent among patients with aerophagia (respectively, 71.1% vs. 58.3%, *p* = 0.006 and 8.3% vs. 3.7%, *p* = 0.025).

### 3.1. Predictors of Aerophagia

**Figure 1:**
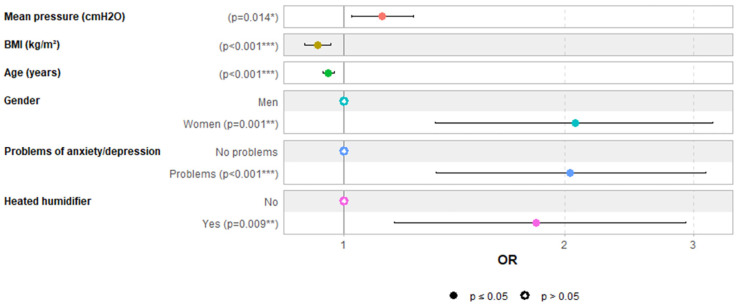
Predictors of aerophagia in multivariable logistic regression analysis. **Note:** each of the six colors is associated with a distinct variable. *** for *p* < 0.001, ** for *p* < 0.01 and * for *p* < 0.05.

Supplementary Files S1 and S2 summarize univariate and multivariable logistic regression analyses evaluating the impact of explanatory variables on aerophagia. Figure 1 depicts the multivariable regression analysis (AUC: 0.72 [0.67; 0.77]). The multivariable logistic regression analysis identified several independent predictors of aerophagia. Higher mean CPAP pressure was significantly associated with an increased risk of aerophagia, with an odds ratio (OR) of 1.13 per cm H_2_O increase (OR: 1.13, 95% CI: 1.03–1.24, *p* = 0.014). Lower BMI was found to be a protective factor (OR: 0.92, 95% CI: 0.89–0.96, *p* < 0.001). Similarly, older age decreased the likelihood of aerophagia by 5% per year (OR: 0.95, 95% CI: 0.94–0.97, *p* < 0.001). Female gender was a strong predictor (OR: 2.07, 95% CI: 1.34–3.19, *p* = 0.001). Psychological factors also played a significant role, as the presence of anxiety or depression doubled the likelihood of aerophagia (OR: 2.04, 95% CI: 1.33–3.11, *p* < 0.001). Finally, using a heated humidifier was associated with a higher risk of aerophagia (OR: 1.83, 95% CI: 1.16–2.89, *p* = 0.009).

### 3.2. Prevalence of Mask-Related Side Effects According to Aerophagia

**Figure 2:**
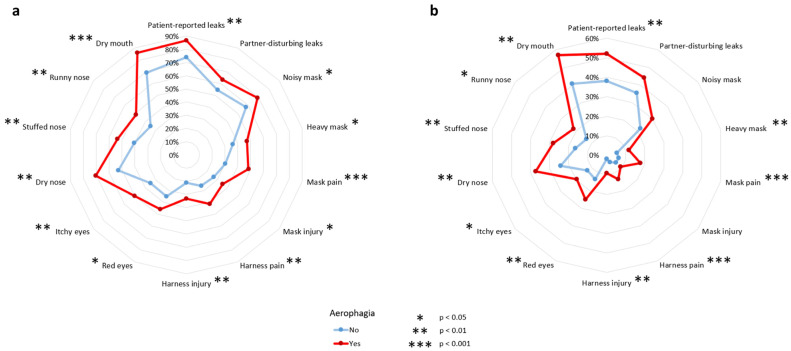
Mask-related side effects according to presence of aerophagia, or not, in patients with a VAS score ≥ 1 (**a**) and VAS score ≥ 5 (**b**).

Figure 2 depicts mask-related side effect frequencies according to aerophagia in subjects with a VAS score ≥ 1 (Figure 2a and Supplementary Materials File S3) and those with a VAS score ≥ 5 (Figure 2b and Supplementary Materials File S4). All mask-related side effects were higher in patients with aerophagia. Patient-reported leaks were higher in patients with aerophagia than in patients without aerophagia (*p* = 0.004 for VAS score ≥ 1 and *p* = 0.005 VAS score ≥ 5). For both VAS score ≥ 1 and VAS score ≥ 5, partner-disturbing leaks did not differ between those with and without aerophagia (*p* = 0.076 and *p* = 0.077, respectively). Dry mouth was more frequent in patients with aerophagia than in patients without aerophagia (*p* < 0.001 for VAS score ≥ 1 and *p* = 0.002 for VAS score ≥ 5).

## 4. Discussion

In this ancillary analysis of the InterfaceVent study, the prevalence of CPAP-related aerophagia was 8.3%, and we identified several clinical and technical factors—particularly higher mean pressure, younger age, lower BMI, female sex, anxiety or depression, and the use of a heated humidifier—as being independently associated with this under-recognized side effect. Aerophagia was also linked to increased mask-related discomfort and reduced CPAP usage, highlighting its potential impact on treatment tolerance.

### 4.1. Overview of Published Evidence on CPAP-Related Aerophagia

To date, only a limited number of studies have investigated CPAP-related aerophagia [4,5,6,7,8,9]. As summarized in Supplementary Materials File S5, these studies are heterogeneous in design, sample size, and populations, with one retrospective cohort, two prospective cohorts, one case–control study, and one randomized crossover trial. Prevalence rates ranged from 7.2% to 27%; notably, our European study found a prevalence of 8.3%, closely matching the 7.2% reported by Fukutome in a Japanese cohort, despite cultural and healthcare system differences.

Despite differences in population characteristics—particularly age, BMI, and CPAP treatment duration—several common predictors have emerged across studies. Higher CPAP pressure has been identified as a risk factor in two studies, including ours [5,6]. Gastroesophageal reflux disease and the use of reflux medications were each implicated in three studies [5,6,7]. Younger age and lower BMI were also associated with aerophagia in both our study and Fukutome’s [5]. However, to date, variables such as gender, anxiety/depression, use of a heated humidifier, or CPAP mode have only been reported in one study. Overall, published evidence remains fragmented, with methodological heterogeneity and limited multivariable adjustment across studies.

Our findings, consistent with Fukutome’s study [5], demonstrate that higher CPAP pressures significantly contribute to aerophagia. This aligns with prior evidence suggesting that lowering pressure levels can effectively reduce aerophagia symptoms. Shirlaw et al. reported that auto-CPAP settings, which reduce median and 95th percentile pressures compared to fixed CPAP settings, significantly alleviated aerophagia symptoms [9]. Similarly, Palot et al. demonstrated that switching from CPAP to bilevel PAP, which provides lower expiratory pressures, significantly reduced aerophagia-related symptoms while maintaining therapy compliance and treatment effectiveness over one year [10]. However, in our study, we found that auto-CPAP did not confer any benefit on aerophagia prevalence. This discrepancy could be due to the contradictory effects of auto-CPAP on pressure, as users tend to experience lower mean pressure but higher 90th/95th percentile mean pressure compared to fixed CPAP users. These findings highlight the importance of personalized CPAP pressure optimization to minimize side effects, like aerophagia, and improve patient adherence. Notably, in our study, CPAP usage was lower in the aerophagia group.

### 4.2. Specific Findings from the Interfacevent Ancillary Analysis

Our dedicated ancillary analysis of the InterfaceVent cohort provides new insights into the clinical and technical factors associated with aerophagia in a large, real-life European population. In contrast to most earlier studies, we systematically assessed a broad spectrum of mask-related side effects and device parameters.

From a clinical perspective, recognition of clinical and technical risk factors may assist clinicians in anticipating and monitoring aerophagia more effectively. Based on our findings, patients who are younger, female, have a lower BMI, report anxiety or depression, or demonstrate poor CPAP adherence should be systematically asked about aerophagia-related symptoms, as these individuals may be particularly vulnerable. Similarly, patients requiring higher therapeutic pressures may benefit from closer follow-up, early troubleshooting, and consideration of pressure-reducing strategies when symptoms occur. Beyond management, this approach may improve long-term CPAP adherence by addressing a frequent and bothersome side effect. Future prospective studies are needed to evaluate whether systematic screening and tailored interventions based on these risk factors can mitigate aerophagia and improve treatment outcomes.

Patients with aerophagia reported more frequent and severe mask-related side effects—especially dry mouth and patient-perceived leaks—suggesting possible overlap between upper airway discomfort and gastrointestinal air intake. While not statistically significant, our study found that facemask use tended to be associated with aerophagia. Facemask use also tended to be associated with higher pressures, less unintentional leaks but more overall leaks, and more frequent use of heated humidifiers or heated circuits. Further research is needed to clarify these findings. Potential hypotheses include (i) higher pressures are associated with greater overall leaks, leading to more side effects that require the use of a heated humidifier or heated tubing, and (ii) less unintentional leakage is associated with aerophagia. This could be because the “externalized/unintentional” leak flow is not “internalized” into the gastrointestinal tract and thus less likely to increase the risk of aerophagia.

### 4.3. Strengths and Limitations of This Study

The major strength of our study lies in the size and comprehensiveness of the InterfaceVent cohort, which includes 1464 well-characterized CPAP-treated patients in a real-life setting. Standardized data collection by trained technicians, use of validated patient-reported outcomes, and robust multivariable statistical modeling enhance the reliability of our findings. To our knowledge, this is the largest prospective European study specifically dedicated to aerophagia.

However, several limitations must be acknowledged. First, aerophagia was assessed through a binary self-reported measure rather than a validated symptom score, limiting granularity. Moreover, the absence of information on whether symptoms were new or pre-existing may have introduced misclassification, thereby reducing reproducibility. Second, not only data on gastroesophageal reflux disease (GERD) or reflux medications but also data on comorbidities including irritable bowel syndrome and history of bowel obstruction was not available in the data set. These comorbidities are important potential confounders. This absence limits the ability to adjust for confounding and must be considered when interpreting our findings. Third, although our cross-sectional design allows for identification of associations, it precludes causal inference. It should be emphasized that this study was not designed to establish causal relationships. Rather, its cross-sectional design was conceived to describe side effects associated with CPAP use. Therefore, any association observed should be interpreted with caution, as they do not imply causality. Because the study did not include an external control group of untreated OSA patients, the attributable risk of CPAP for aerophagia could not be estimated. In addition, some between-group differences, such as BMI or ESS, reached statistical significance but should be interpreted with caution regarding their clinical relevance. Fourth, patients with pre-existing gastrointestinal conditions were not excluded, as the primary objective of this study was to assess CPAP- and mask-related side effects in a real-life cohort. Future studies should consider excluding such patients to better differentiate CPAP-induced aerophagia from pre-existing digestive symptoms. Fifth, information on medication use was not collected. Drugs known to influence gastrointestinal symptoms—including GERD therapies, prokinetics, anticholinergics, alpha-glucosidase inhibitors, and osmotic laxatives—could therefore not be adjusted for in our analyses. This omission introduces potential bias and residual confounding, which should be considered when interpreting the findings. The multivariable analysis did not include important potential confounders such as GERD, gastrointestinal medications, or other comorbidities, which further limits the reliability of the reported associations. Finally, as with any real-life study, unmeasured confounding may persist despite adjustment.

## 5. Conclusions

This ancillary analysis of the InterfaceVent study contributes to a better understanding of CPAP-related aerophagia by identifying associated clinical and technical factors in a large, real-life cohort. These findings apply specifically to patients with obstructive sleep apnea treated with CPAP and should not be generalized beyond this population. Importantly, given the cross-sectional design, the associations observed cannot be interpreted as causal relationships. Nevertheless, our findings highlight the need for systematic screening of aerophagia during CPAP follow-up, particularly in high-risk subgroups such as younger, non-obese women or patients exposed to higher pressure levels. Further prospective studies, ideally incorporating validated symptom scales and exploring interventions such as pressure adjustment, are warranted to improve patient comfort and long-term adherence.

## Figures and Tables

**Table 1:** Population characteristics.

		Aerophagia		
Variable	Whole Population *n* = 1461	No *n* = 1 340	Yes *n* = 121	*p*-Value
**Age (years)**	67.0 (60.0; 74.0)	67.0 (61.0; 74.0)	63.0 (55.0; 72.0)	**<0.001 ^1^**
**Age (years), *n* (%)**				**0.002 ^2^**
[27, 63]	524 (35.9)	463 (34.6)	61 (50.4)	
(63, 71]	466 (31.9)	437 (32.6)	29 (24.0)	
(71, 95]	471 (32.2)	440 (32.8)	31 (25.6)	
**BMI (kg/m^2^)**	30.8 (27.6; 34.9)	30.9 (27.7; 35.1)	30.1 (27.1; 33.7)	**0.024 ^1^**
**BMI (kg/m^2^), *n* (%)**				0.053 ^2^
<25 kg/m^2^	128 (9.8)	110 (9.2)	18 (16.4)	
25 to 30 kg/m^2^	442 (33.8)	408 (34.1)	34 (30.9)	
≥30 kg/m^2^	737 (56.4)	679 (56.7)	58 (52.7)	
**Gender (women), *n* (%)**	404 (27.7)	354 (26.4)	50 (41.3)	**<0.001 ^2^**
**Diagnostic AHI (events/h)**	39.0 (31.0; 56.0)	39.0 (31.0; 56.0)	36.0 (30.0; 52.8)	0.079 ^1^
**Diagnostic AHI (events/h), *n* (%)**				**0.001 ^2^**
[15, 30]	180 (13.8)	153 (12.9)	27 (23.7)	
More than 30	1 125 (86.2)	1 038 (87.2)	87 (76.3)	
**Active smokers, *n* (%)**	171 (11.9)	152 (11.5)	19 (16.1)	0.143 ^2^
**Active workers, *n* (%)**	291 (20.6)	248 (19.1)	43 (36.8)	**<0.001 ^2^**
**Presence of partner, *n* (%)**	1 035 (72.2)	950 (72.4)	85 (70.3)	0.621 ^2^
**ESS (0–24 score)**	5.0 (3.0; 9.0)	5.0 (3.0; 8.0)	7.0 (4.0; 10.0)	**<0.001 ^1^**
**RES, *n* (%)**	236 (16.2)	209 (15.6)	27 (22.3)	0.055 ^2^
**EQ-5D-3L**				
Problems with mobility, *n* (%)	340 (24.2)	317 (24.6)	23 (19.8)	0.251 ^2^
Problems with self-care, *n* (%)	84 (6.0)	78 (6.1)	6 (5.1)	0.667 ^2^
Problems with usual activities, *n* (%)	277 (19.8)	254 (19.8)	23 (19.5)	0.936 ^2^
Problems of pain/discomfort, *n* (%)	818 (58.0)	746 (57.7)	72 (61.5)	0.420 ^2^
Problems of anxiety/depression, *n* (%)	555 (39.4)	486 (37.6)	69 (58.5)	**<0.001 ^2^**
EQ-5D-3L health VAS (0–100 score)	69.9 (50.5; 80.2)	69.9 (50.5; 80.1)	70.1 (50.5; 80.4)	0.849 ^1^
**CPAP usage (h/day)**	6.8 (5.5; 7.8)	6.8 (5.5; 7.8)	6.4 (5.3; 7.3)	**0.001 ^1^**
**CPAP usage (h/day)**				**0.004 ^2^**
[0, 4)	113 (7.7)	100 (7.5)	13 (10.7)	
[4, 6)	347 (23.8)	312 (23.3)	35 (28.9)	
[6, 8)	682 (46.7)	620 (46.3)	62 (51.2)	
≥8	319 (21.8)	308 (23.0)	11 (9.1)	
**Non-adherence, *n* (%)**	113 (7.7)	100 (7.5)	13 (10.7)	0.196 ^2^
**Current AHI_flow_ (events/h)**	1.9 (0.9; 4.0)	2.0 (1.0; 4.0)	1.8 (0.8; 3.0)	0.101 ^1^
**Treatment duration (years)**	4.5 (2.1; 9.8)	4.6 (2.1; 9.9)	3.7 (1.5; 8.5)	**0.040 ^1^**
**Mean pressure (**cm H_2_O**)**	8.2 (6.7; 10.0)	8.1 (6.6; 10.0)	8.5 (7.2; 10.1)	0.111 ^1^
**Mean pressure (**cm H_2_O**), *n* (%)**				**0.018 ^2^**
[4, 6)	183 (12.7)	175 (13.2)	8 (6.7)	
[6, 8)	449 (31.2)	410 (31.0)	39 (32.8)	
[8, 10)	420 (29.2)	385 (29.1)	35 (29.4)	
[10, 12)	304 (21.1)	269 (20.4)	35 (29.4)	
≥12	85 (5.9)	83 (6.3)	2 (1.7)	
**90th/95th pressure (**cm H_2_O**)**	10.0 (8.0; 11.8)	10.0 (8.0; 11.8)	10.3 (9.0; 11.9)	0.062 ^1^
**90th/95th pressure (**cm H_2_O**), *n* (%)**				0.167 ^3^
[4, 6)	32 (2.3)	32 (2.5)	0 (0.0)	
[6, 8)	233 (16.6)	217 (16.8)	16 (13.9)	
[8, 10)	375 (26.6)	348 (26.9)	27 (23.5)	
[10, 12)	516 (36.7)	464 (35.9)	52 (45.2)	
≥12	252 (17.9)	232 (17.9)	20 (17.4)	
**Fixed pressure, *n* (%)**	190 (13.0)	177 (13.2)	13 (10.7)	0.438 ^2^
**Comfort mode, *n* (%)**	232 (15.9)	208 (15.5)	24 (19.8)	0.214 ^2^
**Heated humidifier, *n* (%)**	867 (59.3)	781 (58.3)	86 (71.1)	**0.006 ^2^**
**Heated breathing tube, *n* (%)**	59 (4.0)	49 (3.7)	10 (8.3)	**0.025 ^3^**
**Type of mask, *n* (%)**				0.331 ^2^
Nasal	795 (54.4)	732 (54.6)	63 (52.1)	
Nasal pillows	250 (17.1)	233 (17.4)	17 (14.1)	
Oronasal	416 (28.5)	375 (28.0)	41 (33.9)	
**Mask availability since 2013, *n* (%)**	601 (41.3)	541 (40.5)	60 (49.6)	0.052 ^2^
**Unintentional leaks (L/min)**	2.5 (0.0; 7.5)	2.5 (0.0; 7.5)	1.2 (0.0; 4.8)	**0.036 ^1^**
**Unintentional large leaks (%)**	0.1 (0.0; 0.9)	0.1 (0.0; 0.9)	0.0 (0.0; 0.6)	0.423 ^1^
**Global leaks (L/min)**	33.0 (27.0; 41.0)	33.0 (27.0; 40.3)	37.0 (33.5; 41.5)	0.295 ^1^
**Global large leaks (%)**	0.9 (0.1; 5.1)	0.9 (0.1; 5.1)	1.0 (0.6; 9.5)	0.299 ^1^

Data are reported as median (IQR), unless otherwise specified. AHI: Apnea–Hypopnea Index; AHI_flow_: AHI reported by device; BMI: Body Mass Index; CPAP: continuous positive airway pressure; ESS: Epworth Sleepiness Scale; *n* = number of patients responding; Non-adherence: CPAP usage under 4 h per day; RES: Residual Excessive Sleepiness (ESS score > 10); VAS: visual analogue scale. Mask availability since 2013 indicates the year when the mask type became available on the French market for prescription. Data are reported as medians and quartiles or numbers and percentages of total as appropriate. ^1^ Wilcoxon rank sum test; ^2^ Pearson’s Chi-squared test; ^3^ Fisher’s exact test. Bolds variables are statistically significant at the 5% threshold.

## Data Availability

The datasets used and/or analyzed during the current study are available from the corresponding author on reasonable request.

## Supplementary Materials

The following supporting information can be downloaded at: https://www.mdpi.com/article/10.3390/jcm14186424/s1, Supplementary File S1: Univariate logistic regression analysis on potential predictors of aerophagia. Supplementary File S2: Multivariable logistic regression analysis on predictors of aerophagia. Supplementary File S3: Mask related side effects according to presence of aerophagia in patient with a VAS score ≥ 1. Supplementary File S4: Mask related side effects according to presence of aerophagia in patient with a VAS score ≥ 5. Supplementary File S5: Literature review of potential factors influencing occurrence of aerophagia.

## Abbreviations

AHI	apnea–hypopnea index
BMI	body mass index
CPAP	continuous positive airway pressure
GERD	gastroesophageal reflux disease
OR	odds ratio
OSA	obstructive sleep apnea
VAS	visual analogue scale
