# Supplementary material for: Positive Airway Pressure-Related Aerophagia in Obstructive Sleep Apnea: Results from the InterfaceVent Real-Life Study
Source: J Clin Med. 2025 Sep 11;14(18):6424. doi: 10.3390/jcm14186424 (PMC12471153; doi:10.3390/jcm14186424)
Supplement: Supplementary file 1 [file jcm-14-06424-s001.zip › jcm-3807302-supplementary.pdf]

Supplementary files

Title:

Continuous positive airway pressure-related aerophagia in obstructive sleep apnea: results from the InterfaceVent real-life study.

Authors:

Celia Vidal, MSc (Stat)[1,2], Jean-Pierre Mallet MD[2,3,4], Sarah Skinner PhD[5], Raphael Gilson[2], Olivier Gaubert[2], Arnaud Prigent[6], Frédéric Gagnadoux[7], Jean-Christina Borel[8], Arnaud Bourdin MD, PhD[2,3,4], Nicolas Molinari PhD[1,2,9], and Dany Jaffuel MD, PhD[2,3,4].

Affiliations:

[1] IDESP, INSERM, Montpellier University, Montpellier, France.

[2] Groupe Adène, Montpellier, France.

[3] Department of Respiratory Diseases, Montpellier University Hospital, Arnaud de Villeneuve Hospital, Montpellier, France.

[4] PhyMedExp (INSERM U 1046, CNRS UMR9214), Montpellier University, Montpellier, France.

[5] CHU Montpellier, Montpellier, France.

[6] Polyclinique Saint-Laurent, Groupe Medical de Pneumologie, Rennes, France.

[7] Department of Respiratory and Sleep Medicine, University Hospital of Angers, Angers, France.

[8] Centre de Pneumologie Henri Bazire, la Sure en Chartreuse, Isère, France.

[9] PreMEdical INRIA, CHU Montpellier, Montpellier, France.

Corresponding author:

Vidal Celia, IDESP, INSERM, Montpellier University, Montpellier, France.

E-mail: c.vidal@groupe-adene.com

Tel: +33681036865

Correspondance:

Celia Vidal: c.vidal@groupe-adene.com; ORCID: 0000-0002-8760-6077

Jean-Pierre Mallet: jp-mallet@chu-montpellier.fr; ORCID: 0000-0001-7769-1717

Sarah Skinner: sarah.skinner@chu-montpellier.fr; ORCID: 0000-0003-1089-723X

Raphaël Gilson: r.gilson@groupe-adene.com; ORCID: 0009-0005-4332-7235

Olivier Gaubert: o.gaubert@groupe-adene.com; ORCID: 0009-0006-6364-6265

Arnaud Prigent: dr.arnaudprigent@gmail.com; ORCID : 0000-0001-6302-1130

Frédéric Gagnadoux: frgagnadoux@chu-angers.fr; ORCID: 0000-0002-4231-5102

Jean-Christian Borel: JCBorel1@gmail.com; ORCID: 0000-0003-4140-6210

Arnaud Bourdin: a-bourdin@chu-montpellier.fr; ORCID: 0000-0002-4645-5209

Nicolas Molinari: nicolas.molinari@inserm.fr; ORCID: 0000-0002-1786-0088

Dany Jaffuel: dany.jaffuel@wanadoo.fr; ORCID: 0000-0002-6964-4766

Supplementary file S1. Univariate logistic regression analysis on potential predictors of aerophagia.

Variable	N	OR[1]	95% CI[1]	p-value
**Age (years)**	1 461	0.97	0.95, 0.98	**<0.001**
**Age (years)**	1 461			**0.003**
[27,63]		—	—	
(63,71]		0.50	0.32, 0.80	
(71,95]		0.53	0.34, 0.84	
**BMI (kg/m²)**	1 307	0.96	0.92, 0.99	**0.014**
**BMI (kg/m²)**	1 307			0.079
< 25 kg/m²		—	—	
25 to 30 kg/m²		0.51	0.28, 0.94	
≥ 30 kg/m²		0.52	0.30, 0.92	
**Gender (women)**	1 461	1.96	1.34, 2.87	**<0.001**
**Diagnostic AHI (events/h)**	1 305	0.99	0.98, 1.00	0.229
**Diagnostic AHI (events/h) (More than 30)**	1 305	0.47	0.30, 0.76	**0.003**
**Active smokers**	1 435	1.47	0.88, 2.47	0.160
**Active workers**	1 415	2.46	1.65, 3.67	**<0.001**
**Presence of partner**	1 434	0.90	0.60, 1.36	0.623
**ESS (0-24 score)**	1 461	1.07	1.03, 1.11	**<0.001**
**RES**	1 461	1.55	0.99, 2.44	0.065
**Problems with mobility**	1 405	0.76	0.47, 1.22	0.241
**Problems with self-care**	1 392	0.83	0.35, 1.95	0.660
**Problems with usual activities**	1 401	0.98	0.61, 1.58	0.936
**Problems of pain/discomfort**	1 410	1.17	0.80, 1.73	0.418
**Problems of anxiety/depression**	1 410	2.34	1.59, 3.43	**<0.001**
**EQ-5D-3L health VAS (0-100 score)**	1 367	1.00	0.99, 1.01	0.824
**CPAP-usage (h/day)**	1 461	0.86	0.78, 0.95	**0.004**
**CPAP-usage (h/day)**	1 461			**0.001**
≥ 8		—	—	
[6,8)		2.80	1.45, 5.39	
[4,6)		3.14	1.57, 6.30	
[0,4)		3.64	1.58, 8.38	
**Non-adherence**	1 461	1.49	0.81, 2.75	0.217
**Current AHI_{flow} (events/h)**	1 457	0.92	0.85, 1.00	**0.020**
**Treatment duration (years)**	1 416	0.96	0.93, 1.00	**0.042**
**Mean pressure (cmH_2O)**	1 441	1.06	0.97, 1.15	0.202
**Mean pressure (cmH_2O)**	1 441			**0.009**
[4,6)		—	—	
[6,8)		2.08	0.95, 4.54	
[8,10)		1.99	0.90, 4.38	
[10,12)		2.85	1.29, 6.28	
≥ 12		0.53	0.11, 2.54	
**90th/95th pressure (cmH_2O)**	1 408	1.08	0.99, 1.17	0.091
**90th/95th pressure (cmH_2O)**	1 408			0.065
≥ 12		—	—	
[10,12)		1.30	0.76, 2.23	
[8,10)		0.90	0.49, 1.64	
[6,8)		0.86	0.43, 1.69	
[4,6)		0.00	0.00, Inf	
**Fixed pressure**	1 460	0.79	0.44, 1.44	0.427
**Comfort mode**	1 461	1.35	0.84, 2.16	0.227
**Heated humidifier**	1 461	1.76	1.17, 2.64	**0.005**
**Heated breathing tube**	1 461	2.37	1.17, 4.81	**0.028**
**Type of mask**	1 461			0.335
Nasal		—	—	
Nasal pillows		0.85	0.49, 1.48	
Oronasal		1.27	0.84, 1.92	
**Mask availability since 2013, n (%)**	1457	1.45	1.00, 2.10	0.053
**Unintentional leaks (l/min)**	881	0.97	0.94, 1.01	0.096
**Unintentional large leaks (%)**	398	0.96	0.86, 1.07	0.347
**Global leaks (l/min)**	148	1.02	0.96, 1.08	0.584
**Global large leaks (%)**	137	1.01	0.93, 1.09	0.881

[1]OR = Odds Ratio, CI = Exact Confidence Interval

Supplementary file S2. Multivariable logistic regression analysis on predictors of aerophagia.

Variable	OR[1]	95% CI[1]	p-value	VIF
**Mean pressure (cmH2O)**	1.13	1.03, 1.24	**0.014**	1.10
**BMI (kg/m²)**	0.92	0.89, 0.96	**<0.001**	1.11
**Age (years)**	0.95	0.94, 0.97	**<0.001**	1.08
**Gender**				1.05
Men	—	—		
Women	2.07	1.34, 3.19	**0.001**	
**Problems of anxiety/depression**				1.04
no problems	—	—		
problems	2.04	1.33, 3.11	**<0.001**	
**Heated humidifier**				1.01
No	—	—		
Yes	1.83	1.16, 2.89	**0.009**	

[1]OR = Odds Ratio, CI = Exact Confidence Interval

Supplementary file S3. Mask related side effects according to presence of aerophagia in patient with a VAS score ≥ 1.

Variable	No aerophagia, N = 1 340	Aerophagia, N = 121	p-value	q-value[1]
**Patient-reported leaks, n (%)**	978 (74.15)	105 (86.78)	**0.002**	**0.004**
**Partner-disturbing leaks, n (%)**	667 (54.49)	69 (63.30)	0.076	0.076
**Noisy mask, n (%)**	762 (57.81)	83 (69.17)	**0.016**	**0.021**
**Heavy mask, n (%)**	476 (35.98)	56 (47.06)	**0.016**	**0.021**
**Mask pain, n (%)**	401 (30.20)	57 (48.31)	**<0.001**	**<0.001**
**Mask injury, n (%)**	354 (26.72)	42 (35.29)	**0.045**	**0.048**
**Harness pain, n (%)**	343 (25.95)	49 (41.18)	**<0.001**	**0.001**
**Harness injury, n (%)**	279 (21.18)	40 (33.33)	**0.002**	**0.004**
**Red eyes, n (%)**	463 (35.02)	55 (45.83)	**0.018**	**0.021**
**Itchy eyes, n (%)**	458 (34.62)	59 (50.00)	**<0.001**	**0.002**
**Dry nose, n (%)**	700 (52.95)	83 (70.34)	**<0.001**	**0.001**
**Stuffed nose, n (%)**	529 (40.41)	65 (53.72)	**0.004**	**0.007**
**Runny nose, n (%)**	458 (34.62)	58 (48.74)	**0.002**	**0.004**
**Dry mouth, n (%)**	917 (69.16)	104 (85.95)	**<0.001**	**<0.001**

[1]Fasle discovery rate correction

Supplementary file S4. Mask related side effects according to presence of aerophagia in patient with a VAS score ≥ 5.

Variable	No aerophagia, N = 1 340	Aerophagia, N = 121	p-value	q-value[1]
**Patient-reported leaks, n (%)**	503 (38.13)	63 (52.07)	**0.003**	**0.005**
**Partner-disturbing leaks, n (%)**	433 (35.38)	48 (44.04)	0.071	0.077
**Noisy mask, n (%)**	291 (22.08)	36 (30.00)	**0.047**	0.055
**Heavy mask, n (%)**	70 (5.29)	14 (11.76)	**0.004**	**0.006**
**Mask pain, n (%)**	83 (6.25)	21 (17.80)	**<0.001**	**<0.001**
**Mask injury, n (%)**	81 (6.11)	11 (9.24)	0.180	0.180
**Harness pain, n (%)**	52 (3.93)	16 (13.45)	**<0.001**	**<0.001**
**Harness injury, n (%)**	25 (1.90)	11 (9.17)	**<0.001**	**0.001**
**Red eyes, n (%)**	180 (13.62)	30 (25.00)	**<0.001**	**0.002**
**Itchy eyes, n (%)**	166 (12.55)	23 (19.49)	**0.032**	**0.041**
**Dry nose, n (%)**	321 (24.28)	44 (37.29)	**0.002**	**0.004**
**Stuffed nose, n (%)**	215 (16.42)	34 (28.10)	**0.001**	**0.003**
**Runny nose, n (%)**	175 (13.23)	26 (21.85)	**0.009**	**0.013**
**Dry mouth, n (%)**	541 (40.80)	69 (57.02)	**<0.001**	**0.002**

[1]Fasle discovery rate correction

Supplementary file S5. Literature review of potential factors influencing occurrence of aerophagia

	Fukutome, 2024	Watson et al., 2008	Shepherd et al., 2013	Shirlaw et al., 2017	Current study
Type of study	Retrospective	Case-control	Prospective	Crossover	Prospective
Number of patients total	753	44	477	56	1461
Number (%) of patients with aerophagia	54 (7,2%)	22 (50%)	130 (27%)	56 (100%)	121 (8,30%)
Demographics					
Male (%)	85.2	50.0	75.0	69.6	58.7
Age (years)	51.4	49.3	54.0 ± 13.0	65. ± 13.0	63.0 (55.0; 72.0)
BMI (kg/m²)	26.2	31.1	34.0 ± 7.0	34.2 ± 6.2	30.1 (27.1; 33.7)
Clinical characteristics					
Diagnostic AHI (events/hr)	50.6	38.3 (31.0; 45.6)	NR	45.0 ± 29.5	36.0 (30.0; 52.8)
GER (%)	25.9	77.3	57.0	18.0	NR
GER medications (%)	NR	45.5	32.0	NR	NR
CPAP treatment					
AHI residual (events/hr)	2.3	NR	14.0 ± 20.0	4.3 (2.9; 7.5)	1.8 (0.8; 3.0)
90th/95th CPAP pressure cmH2O	9.4	10.5 (9.3; 11.8)	11.0 ± 2.0	14.0 (12.0; 17.9)	10.3 (9.0; 11.9)
CPAP-usage ≥4h (%)	87.0	NR	NR	92	89.3
CPAP usage duration (yr)	6.3	NR	NR	0.9 (0.3; 3.7)	3.7 (1.5; 8.5)
Nasal mask (%)	94.4	NR	NR	30.4	52.1
Predictors of aerophagia					
GER	✓	✓	✓	NA	NR
GER medications	NR	✓	✓	NR	NR
Lower age	✓	✘	✘	NA	✓
Lower BMI	✓	✘	✘	NA	✓
No tobacco	NR	✓	NR	NA	NR
Oxygen	NR	✓	NR	NR	NR
Higher CPAP pressure	✓	✘	✘	NA	✓
CPAP (vs. APAP)	NR	NR	NR	✓	NR
Women	✘	✘	✘	NA	✓

		Anxiety/depression	NR	NR	NR	NA	✓
		Heated humidifier	NR	NR	NR	NR	✓

AHI: Apnea–Hypopnea Index; BMI: Body Mass Index; CPAP: Continuous Positive Airway Pressure; GER: Gastroesophageal reflux disease; APAP: Autotitrating Positive Airway Pressure; NA : Not Applicable; NR: Not Reported in the study; ✓: Predictor of aerophagia, ✘: Not a predictor of aerophagia.
